# Multimodal Detection of Subclinical Cardiovascular Disease in Chronic Kidney Disease

**DOI:** 10.1159/crm/abtag004

**Published:** 2026-08-31

**Authors:** Inhae Baek, Purvi Parwani, Safwan Gaznabi, Dmitry Abramov, Anas Alani

**Affiliations:** Cardiology, Loma Linda University Medical Center, Loma Linda, CA, USA

**Keywords:** Subclinical, Cardiovascular disease, Chronic kidney disease, Cardiorenal syndrome

## Abstract

**Background:**

Cardiovascular disease (CVD) is the leading cause of mortality among individuals with chronic kidney disease (CKD) and is driven by chronic inflammation, oxidative stress, vascular calcification, accumulation of uremic toxins, ventricular remodeling, and microvascular dysfunction. Subclinical CVD is highly prevalent in CKD and often remains unrecognized until advanced stages, contributing to delayed intervention and increased mortality.

**Summary:**

Early detection of subclinical CVD in CKD is critical to prevent disease progression and reduce adverse outcomes. Advances in noninvasive imaging modalities – including echocardiography, coronary computed tomography angiography, coronary artery calcium (CAC) scoring, and cardiac magnetic resonance imaging – enable sensitive identification of early cardiac and vascular abnormalities. In parallel, circulating and urinary biomarkers such as C-reactive protein, high-sensitivity troponin (hsT), N-terminal pro-brain natriuretic peptide, interleukin-6 (IL-6), tumor necrosis factor-alpha (TNF-α), fibroblast growth factor-23, and urine albumin-to-creatinine ratio provide insight into underlying pathophysiological mechanisms and support risk stratification and personalized management.

**Key Messages:**

Subclinical CVD is common yet underdiagnosed in CKD, necessitating improved screening strategies. Integration of advanced imaging techniques with biomarker profiling offers a promising approach for early detection and individualized care. Continued innovation and implementation of these tools are essential to closing the cardiovascular mortality gap in this high-risk population.

## Introduction

Cardiovascular disease (CVD) accounts for the highest morbidity and mortality among patients with chronic kidney disease (CKD). The burden of CVD in patients with CKD is substantial, with a significantly increased risk of cardiovascular death compared to the general population [1]. Furthermore, individuals with comorbid CKD have not experienced the same improvements in CVD mortality over the prior 2 decades as patients without comorbid renal disease [2]. CKD accelerates CVD through systemic inflammation, atherogenesis, microvascular dysfunction, and compounds traditional risk factors such as hypertension, obesity, dyslipidemia, and diabetes [3, 4]. These processes precipitate subclinical pathologies such as left ventricular hypertrophy, myocardial fibrosis, and vascular calcification, which may result in heart failure, fatal arrhythmias, myocardial infarction, and sudden cardiac death [5, 6]. Subclinical CVD is prevalent yet underdiagnosed in CKD, which leads to delayed intervention and high morbidity and mortality. However, advances in cardiac imaging allow the detection of subtle structural and functional changes prior to clinical manifestations [7, 8]. Simultaneously, cardiac biomarkers are increasingly used to assess cardiovascular risk and subclinical disease in CKD, as these circulating biomarkers are often increased before symptoms occur [9, 10]. Integrating these modalities offers early recognition, coordinated management, and improved outcomes through multidisciplinary management in CKD-associated CVD, which not only decreases cardiovascular mortality but also preserves kidney function and quality of life [11].

## Pathophysiological Links between Subclinical CVD in CKD

The relationship between CKD and subclinical CVD is multifaceted. The bidirectional pathophysiology includes renal dysfunction causing cardiac injury through volume overload, neurohormonal activation, anemia, systemic inflammation, accelerated coronary disease, and sodium retention, while cardiac dysfunction reciprocally worsens renal function [12]. In addition to promoting epicardial coronary atherosclerosis, CKD is increasingly recognized to drive coronary microvascular dysfunction and small-vessel ischemia, which together contribute to the high burden of subclinical CVD observed in this population [13–15]. Understanding these links is fundamental for developing management strategies for patients with both conditions.

### Accelerated Atherosclerosis and Epicardial CAD

CKD is associated with an early and disproportionate burden of epicardial coronary artery disease that appears early in the course of kidney dysfunction and contributes substantially to premature cardiovascular morbidity and mortality [12, 16–18]. Patients with CKD have a higher prevalence and extent of coronary artery calcification and obstructive CAD than individuals without CKD, translating into markedly elevated rates of myocardial infarction and cardiovascular mortality [12, 16, 19]. In addition to a greater plaque burden, coronary lesions in CKD are often more calcified and diffusely distributed, reflecting a vascular milieu shaped by disordered mineral metabolism, chronic inflammation, oxidative stress, and uremic toxins [12, 14, 18]. Clinically, this accelerated epicardial disease underlies a high burden of subclinical ischemia and adverse events, even in patients receiving standard secondary prevention [12, 17, 19].

### Microvascular Disease and Subclinical Ischemia

Beyond epicardial stenosis, CKD is increasingly recognized as a systemic microvascular disorder involving the coronary circulation. Generalized microvascular rarefaction, endothelial dysfunction, and small-vessel remodeling in CKD reduces vasodilatory reserve and predispose to tissue ischemia in the absence of obstructive epicardial CAD. [13, 20]. CKD induces a systemic microangiopathy with loss of coherent capillary networks, reduced perfusion, and large avascular areas in the skeletal muscle and myocardium, supporting a direct toxic effect of the uremic milieu on the microcirculation [20]. Human studies similarly show reduced microvascular density and structural abnormalities of small vessels in multiple vascular beds in CKD, often preceding or exceeding overt macrovascular disease [13, 15]. Within the coronary circulation, impaired coronary flow reserve is frequently observed in CKD patients even when epicardial coronary arteries lack flow-limiting stenoses, indicating coronary microvascular dysfunction as a distinct ischemic substrate [15, 21]. Importantly, depressed coronary flow reserve in CKD is strongly associated with adverse outcomes, including cardiac death and heart-failure hospitalization, independent of traditional risk factors and angiographic epicardial disease severity [21]. Collectively, these data support microvascular rarefaction and dysfunction as key drivers of subclinical ischemia in CKD and a critical target for improved cardiovascular risk stratification in this population [13, 21].

### Vascular Calcification and Arterial Stiffness

Vascular calcification is an early and prevalent phenomenon in CKD, leading to increased arterial stiffness and a higher cardiovascular event rate. Vascular calcification is linked to the dysregulation of calcium and phosphate metabolism, leading to increased serum phosphate and subsequent deposition of calcium phosphate crystals, in the form of hydroxyapatite, in both intimal and medial layers of blood vessels [14, 22, 23]. CKD drives vascular smooth muscle cells to differentiate into osteoblast-like cells, promoting inappropriate mineral deposition and vascular ossification. The subsequent arterial stiffness leads to a loss of vascular compliance, increased shear stress and systolic blood pressure, and reduced coronary perfusion [24]. Intimal calcification predominantly manifests as plaque rupture and acute ischemic events, whereas medial calcification promotes arterial stiffness, elevated systolic pressure, and subsequent left ventricular hypertrophy (LVH) [25]. Importantly, vascular calcification and arterial stiffness are now recognized as therapeutic targets in CKD-mineral-bone disorder, with interventions including phosphate binders and vitamin D analogs, aiming to modulate the mineral-bone axis and slow progression [23, 26]. Vascular calcification is now recognized as a nontraditional cardiovascular risk factor in patients with CKD, yet management remains challenging, and these pathologies often progress silently, underscoring the need for aggressive risk factor modification and routine cardiovascular assessment in CKD patients [23].

### Left Ventricular Hypertrophy and Remodeling

Left ventricular hypertrophy and myocardial remodeling represent cardiac manifestations in the progression of CKD-related CVD and are strongly associated with adverse outcomes [27–29]. The prevalence of LVH rises with advancing CKD stage, exceeding 70% among patients with end-stage renal disease [30]. Both concentric and eccentric patterns of hypertrophy can occur, driven by chronic pressure overload from arterial hypertension and stiffness, as well as persistent volume overload due to sodium and fluid retention [27, 31, 32]. The pathogenesis of LVH in CKD also involves non-hemodynamic factors, such as chronic inflammation, oxidative stress, activation of the renin-angiotensin-aldosterone system, and exposure to uremic toxins, which promote myocyte hypertrophy and myocardial fibrosis [5, 28, 33]. Myocardial fibrosis, detectable using advanced imaging modalities like cardiac magnetic resonance (CMR), impairs diastolic and systolic function and predisposes to arrhythmias, heart failure, and sudden cardiac death [29, 32]. LVH and remodeling frequently develop in CKD, and their prognostic value is further underscored by worsening outcomes even in the absence of overt cardiac symptoms [29, 32, 34]. Echocardiography offers sensitive tools for early recognition of LVH and subtle functional decline before ejection fraction drops [28]. Early identification allows for therapeutic interventions targeting underlying mechanisms, which can both mitigate risk and slow progression [5, 12, 27, 34]. Thus, LVH and cardiac remodeling serve as crucial indicators of CKD-related cardiovascular risk and highlight the need for ongoing, multimodal screening in these patients [5, 27, 33, 34].

### Role of Inflammation, Uremic Toxins, and Oxidative Stress

Chronic inflammation is a predominant feature of CKD that accelerates CVD. The retention of uremic toxins, persistent inflammation, and heightened oxidative stress drive the early onset and progression of subclinical CVD [35, 36]. CKD is characterized by a systemic inflammatory state and sustained elevation of pro-inflammatory cytokines – including tumor necrosis factor-alpha (TNF-α), interleukin-6 (IL-6), and C-reactive protein (CRP), that contribute directly to early vascular dysfunction, myocardial remodeling, and accelerated atherogenesis [37, 38]. This inflammatory milieu is partly fueled by the accumulation of uremic toxins – metabolic byproducts normally excreted by healthy kidneys but retained in CKD due to reduced estimated glomerular filtration rate (eGFR) [39, 40]. These toxins not only activate immune cells but also induce endothelial dysfunction, which drives the early onset and progression of subclinical CVD [37, 39, 41].

Oxidative stress is a hallmark feature of CKD and acts in concert with inflammation and uremic toxins to accelerate cardiovascular decline [42, 43]. Notably, uremic toxins stimulate excess generation of ROS, which then further amplify the expression of inflammatory cytokines [40, 41]. This oxidative burden sets the stage for early onset hypertension, left ventricular hypertrophy, and myocardial fibrosis – established features of subclinical CVD [41, 43, 44]. Simultaneously, the chronic inflammatory environment perpetuates a vicious cycle, as activated immune cells and vascular tissues themselves release additional ROS and perpetuate cytokine signaling [36, 42]. Altogether, the interplay of chronic inflammation, uremic toxin retention, and oxidative stress uniquely predisposes CKD patients to accelerated cardiovascular damage often well before overt symptoms, highlighting the necessity of early, multimodal detection and targeted intervention of this population [36, 40].

## Imaging Modalities for Detection of Subclinical CVD

Cardiovascular imaging is central to detecting subclinical disease in CKD, where conventional symptoms and signs may be absent until advanced dysfunction occurs. Advances in imaging techniques have considerably enhanced the detection of subclinical CVD in CKD patients (Table 1) [1, 7].

### Echocardiography

Echocardiography remains a mainstay imaging modality in the early detection and assessment of cardiovascular abnormalities in chronic kidney disease, offering a noninvasive and widely accessible imaging modality capable of real-time evaluation of cardiac structure and function [45]. CKD patients are at increased risk for developing subclinical cardiac dysfunction, including left ventricular hypertrophy, diastolic dysfunction, and subtle changes in myocardial mechanics, all of which precede overt heart failure and ischemic events [46, 47]. Advanced echocardiographic techniques, such as tissue Doppler imaging, strain imaging including global longitudinal strain (GLS), and three-dimensional echocardiography, have substantially improved sensitivity to subclinical changes, allowing for earlier identification of subclinical myocardial dysfunction before a decline in ejection fraction or the onset of symptoms [45]. GLS in particular has emerged as a powerful predictor of adverse outcomes in CKD, surpassing conventional parameters such as ejection fraction and diastolic dysfunction, in detecting early myocardial dysfunction [48]. Patients with impaired GLS and preserved ejection fraction have markedly increased risk for heart failure hospitalization and mortality [49]. Serial echocardiograms can also monitor disease progression, detect subtle cardiac remodeling over time, and inform therapeutic decision-making – especially important for patient populations undergoing dialysis or progressing to end-stage renal disease [27, 47]. Recent studies also highlight how routine and advanced echocardiography can quantify parameters such as left atrial volume, E/e’ ratios (markers of diastolic pressures), pulmonary pressures, and ventricular geometry – all providing independent prognostic value beyond traditional cardiovascular risk factors [16, 50]. By enabling early identification and longitudinal monitoring of subclinical cardiac impairment in CKD, echocardiography fosters timely intervention and risk stratification, ultimately playing a central role in reducing cardiovascular morbidity in this vulnerable population [27, 45, 51]. Despite its strengths, echocardiographic assessment in CKD patients can be limited by poor acoustic windows, alterations in volume status, and confounding effects of hypertension and anemia, prompting the need for complementary imaging modalities in certain cases [51].

Beyond traditional echocardiography, three-dimensional (3D) echocardiography provides more accurate and reproducible quantification of left ventricular volumes, ejection fraction, and mass compared with two-dimensional methods, reducing geometric assumptions that can underestimate remodeling in hypertrophied or dilated ventricles. This approach has proven particularly valuable for serial monitoring in CKD, where progressive structural changes and fluctuations in volume status are common [52]. Stress echocardiography, including dobutamine and exercise-based protocols, offers a safe, noninvasive method for assessing myocardial ischemia, contractile reserve, and viability, particularly in patients being evaluated for renal transplantation or with limited exercise capacity [53]. In this population, stress echocardiography aids in detecting silent ischemia and evaluating perioperative cardiovascular risk [54]. Integrating these modalities alongside conventional echocardiography provides a more comprehensive evaluation of both structural and functional cardiac abnormalities across the CKD spectrum.

### Cardiac Magnetic Resonance Imaging

Cardiac magnetic resonance imaging (CMR) is increasingly recognized for its role in assessing myocardial structure and function in CKD patients, distinguishing itself with high spatial resolution, reproducibility, and unique tissue characterization abilities [6, 55, 56]. The principal clinical value of CMR lies in its ability to detect and quantify myocardial fibrosis, edema, and subtle changes in cardiac function, all of which are prevalent and prognostically significant in CKD – even during subclinical phases [6, 16]. Some CMR techniques also enable noninvasive identification of diffuse myocardial fibrosis even without the use of contrast agents, a critical advantage given the risk of nephrogenic systemic fibrosis (NSF) in advanced CKD [56, 57]. It should also be noted that newer generation gadolinium agents (groups II and III) have been less associated with NSF [58]. Cardiac strain with feature tracking, a technique that measures the heart’s shortening and lengthening in different directions in order to measure myocardial deformation, reveals myocardial abnormality and cardiac remodeling early in CKD progression, preceding detectable reductions in ejection fraction and clinical heart failure [43]. CMR with the use of gadolinium-based contrast agents is used for the detection of focal myocardial fibrosis [6]. Late gadolinium enhancement imaging is used to detect not only silent myocardial infarction but also diffuse enhancement consistent with interstitial fibrosis, two common co-existing conditions in patients with CKD [6, 7]. Native T1 mapping, a measure of longitudinal myocardial relaxation time, is a highly sensitive for myocardial edema, ischemia, and fibrosis whereas T2 mapping, a measure of transverse myocardial relaxation time, specifically highlights increased myocardial fluid, or edema [6, 7, 57, 59]. CMR and its biomarkers, although not as readily available as echocardiography, aids in the early identification and prognostication of cardiomyopathy in CKD, facilitating timely intervention and improved outcomes [6].

### Coronary Artery Calcium and Coronary Computed Tomography Angiography

Coronary computed tomography angiography (CCTA) and coronary artery calcium (CAC) scoring have become essential tools for identifying coronary artery disease as patients with kidney disease have a higher burden of vascular and valvular calcifications [7]. Various CT techniques provide critical insights into cardiovascular risk stratification.

#### Coronary Artery Calcium Scoring

CAC scoring is a non-invasive method that quantifies the amount of calcified plaque in the coronary arteries, a pathologic process accelerated in CKD due to disturbances in mineral metabolism, chronic inflammation, and vascular calcification [60, 61]. Elevated CAC scores are common in early CKD, which provides evidence of atherosclerotic burden before symptoms manifest, and may be used to predict future coronary events, while also refining cardiovascular risk stratification in CKD populations where traditional models may underestimate subclinical disease burden [62, 63]. The utility of CAC scoring exists not only in the prognostication of CVD in CKD but also enables timely intervention with medical therapies such as statins, antiplatelet agents, and mineral management, such as hypercalcemia and hyperphosphatemia [60, 61]. Recent improvements in scan resolution, dual-energy technologies, and standardized scoring protocols have improved the sensitivity and specificity of CAC measurements in CKD, while lower radiation doses have made routine screening increasingly feasible [22, 60]. However, limitations exist for advanced CKD or dialysis patients with extensive vascular calcification, and interpretation must account for age, race, and comorbid disease [61].

#### Coronary CT Angiography

The role of coronary CT angiography (CCTA) as a complementary or alternative imaging modality to CAC scoring has expanded significantly, particularly for the evaluation of overall coronary anatomy in CKD [64]. Unlike CAC scoring, which quantifies only calcified atherosclerotic burden, CCTA provides comprehensive visualization of both calcified and noncalcified plaques, enabling assessment of total plaque burden, localization, and degree of luminal stenosis [65]. The high negative predictive value of CCTA reduces unnecessary invasive interventions such as coronary angiograms while also identifying vulnerable plaques before acute syndromes arise [65–67]. CCTA is particularly useful in CKD patients with ambiguous CAC scores or extensive medial calcification, where standard calcium scoring is unable to accurately measure true disease burden [68, 69]. Drawbacks of CCTA over CAC include the use of contrast, increased cost, and processing time. However, contemporary studies suggest that the incidence of contrast-associated AKI after CCTA is low and comparable to non-contrast CT when guideline-directed hydration, contrast minimization, and other preventive measures are applied, supporting appropriate use of CCTA in CKD rather than reflexive avoidance based solely on renal concerns [70–72]. Accurate interpretation is also contingent upon high-quality imaging, which may be compromised in morbid obesity, tachycardia or arrhythmias, respiratory motion, or extensive coronary disease [65].

CCTA has evolved beyond luminal stenosis assessment to become a comprehensive modality for plaque characterization and inflammation imaging. In CKD, where vascular calcification and mixed atherosclerotic plaques are prevalent, CCTA enables quantitative analysis of plaque burden and composition, identifying high-risk features such as low-attenuation plaque, positive remodeling, and spotty calcification that are associated with future cardiac events [73]. The emergence of photon-counting CT has further enhanced spatial resolution and spectral differentiation, improving visualization of microcalcifications and low-attenuation plaque while enabling lower iodine dose – advantages that are particularly relevant in CKD [74]. In addition, CT-derived biomarkers such as the perivascular fat attenuation index (FAI) offer a noninvasive means of detecting coronary inflammation and refining risk assessment beyond anatomy alone [75].

#### Clinical Guidelines and Imaging Safety in CKD

Recent updates in international practice guidelines further support the safe and proactive use of cardiac imaging in CKD, emphasizing both comprehensive cardiovascular risk assessment and modern contrast safety standards. The approach to early detection and risk stratification of CVD in CKD should be viewed within the framework of recent guideline recommendations. The Kidney Disease: Improving Global Outcomes (KDIGO) 2024 Clinical Practice Guideline for the Evaluation and Management of CKD emphasizes that cardiovascular risk assessment and mitigation must be integral to CKD care across all stages [76]. KDIGO 2024 advocates for the incorporation of eGFR and albuminuria into ASCVD risk estimation, periodic reassessment of subclinical cardiovascular injury, and timely initiation of evidence-based therapies, including blood pressure optimization, lipid management, and SGLT2 inhibitor or GLP-1 receptor agonist therapy [76, 77]. These recommendations reinforce the role of imaging and biomarkers discussed in this review as complementary tools to guide individualized preventive strategies.

In parallel, the American College of Radiology’s (ACR) Manual on Contrast Media (2023) and the ACR/National Kidney Foundation (NKF) Consensus Statement on the Use of Iodinated and Gadolinium-Based Contrast Agents in Patients with Kidney Disease highlight that modern low-osmolar iodinated contrast and Group II gadolinium agents carry an extremely low risk of nephrotoxicity or NSF, even in advanced CKD [58]. These documents clarify that clinically indicated contrast-enhanced CT or CMR studies should not be withheld solely on the basis of CKD, provided that appropriate hydration and avoidance of other nephrotoxins are ensured [58, 78]. Incorporating these updated safety standards enables broader and safer application of cardiac CT and MRI in the subclinical CVD assessment of CKD patients [58].

## Biomarkers

The subtle nature of cardiovascular pathology in CKD requires sensitive and specific tools for early recognition. Biomarkers have become essential tools in the identification of CVD in CKD, which allow the assessment of ongoing cardiovascular injury, inflammation, and maladaptive remodeling often before clinical events or overt dysfunction are apparent [79]. The challenge in CKD is compounded by the effects of kidney impairment on biomarker metabolism and clearance, and therefore requires nuanced interpretation of both traditional and novel markers in this high-risk population [10].

### Cardiac Injury Markers

High-sensitivity troponins (hsT) are markers for myocardial injury, and B-type natriuretic peptide (proBNP) – released by ventricular myocytes in response to volume and pressure loading – act as markers for congestive cardiac states. HsT and proBNP are established biomarkers that are used to identify subclinical myocardial injury and predict cardiovascular events [10]. Despite chronically elevated hsT levels due to reduced renal clearance, these baseline elevations and subtle dynamic changes remain significant predictors of cardiovascular morbidity and mortality even in the absence of acute coronary syndrome, and may reflect subclinical myocyte injury and cardiac remodeling [80]. Persistently elevated proBNP also occurs in CKD due to reduced excretion, even in the absence of overt heart failure. Nonetheless, elevations in proBNP often depict volume overload and myocardial stress in the setting of CKD and correlate with the risk of left ventricular dysfunction, hospitalization, and mortality among CKD patients [79]. Serial trend analysis is often necessary for optimal interpretation of both hsT and proBNP in varying stages of kidney impairment [81].

### Urine Albumin-to-Creatinine Ratio

Urine albumin-to-creatinine ratio (UACR) is an inexpensive, widely available biomarker used in the identification of subclinical CVD in CKD, integrating information on glomerular permeability, microvascular damage, and systemic endothelial dysfunction [82]. Albuminuria, defined as UACR 30–300 mg/g, complements eGFR in CKD staging and refines cardiovascular risk stratification. Beyond categorical cutoffs, even UACR levels within the conventionally normal range carry a graded increase in all-cause and cardiovascular mortality, supporting its use as a continuous prognostic marker of subclinical CVD burden rather than a binary diagnostic threshold [83, 84]. Albuminuria is interpreted as a marker of generalized vascular and renal injury, and is independently associated with adverse outcomes across heart failure and CKD populations, even after adjustment for creatinine, eGFR, and natriuretic peptides [85]. Major society guidelines now regard UACR, alongside eGFR, as a core axis for CKD classification and recommend at least annual UACR testing in adults with CKD, diabetes, or hypertension, with more frequent monitoring in those with established albuminuria to guide the intensity of renin-angiotensin system blockade and SGLT2 inhibitor therapy [82, 83, 85]. Expert consensus statements also highlight that any persistent elevation of UACR above 30 mg/g, irrespective of eGFR, should prompt systematic assessment for subclinical CVD and heart failure, given its strong and independent association with atherosclerotic and heart failure-related events [83, 86, 87]. Integrating UACR offers a multimodal strategy in which albuminuria serves as a high-yield biomarker to flag patients who warrant more rigorous evaluation and preventive therapy [84, 85, 88].

### Inflammatory and Oxidative Stress Markers

Chronic inflammation is a cardinal feature of CKD and an important driver of its accelerated cardiovascular burden [89]. Inflammatory markers such as CRP, IL-6, and TNF-α are common biomarkers utilized to predict cardiovascular events in CKD patients [90, 91]. Elevated levels of CRP, the most established inflammatory marker of acute systemic inflammation, strongly predict atherosclerotic progression, myocardial infarction, and all-cause mortality [91]. Patients on hemodialysis typically have baseline elevations in inflammatory cytokines IL-6 and TNF-α, likely due to ineffective clearance. These markers correlate with vascular dysfunction and cardiovascular mortality, and persistent marker elevation portends a substantially higher risk for future events [10, 35, 92].

### Novel Biomarkers

In the last several years, there has been an emergence of promising novel biomarkers, many of which are being studied to fill the gaps left by traditional tools in the early detection and risk assessment of CVD in CKD. Fibroblast growth factor-23, a marker of phosphate metabolism and mineral-bone disease, has been shown to predict left ventricular hypertrophy, heart failure, and mortality among CKD cohorts [79]. Soluble ST2 (sST2), GDF-15, and galectin-3, markers reflecting myocardial fibrosis and tissue stress, are steadily gaining ground for cardiac risk prognostication in both heart disease and CKD [3, 9, 80, 93]. Neutrophil gelatinase-associated lipocalin and kidney injury molecule-1 (KIM-1), urinary biomarkers initially identified as markers of acute kidney injury (AKI) and progression of CKD, have now been observed that their elevations may be associated with an increase in atherosclerotic cardiovascular events, heart failure, and all-cause mortality [94]. Taken together, the nuanced interpretation of cardiac injury markers, inflammatory cytokines, and a new generation of novel biomarkers offers unprecedented potential for personalized treatment strategies in CKD care. With the influence of renal impairment on biomarker kinetics in the setting of chronic inflammation, there is a need for serial and trend-based biomarker assessment rather than static readings (Table 2) [10, 80].

## Conclusion

The identification and management of subclinical CVD in CKD is a pressing challenge that demands a multifaceted and integrative approach. CVD burdens in patients with kidney disease have contributed to a disproportionate and rising mortality rate among affected populations. This review highlights how CKD-specific factors, such as chronic systemic inflammation, oxidative stress, retention of uremic toxins, vascular and ventricular remodeling, are central to the pathogenesis of silent but progressive cardiovascular injury. Early pathologic changes such as vascular calcification, arterial stiffness, left ventricular hypertrophy, and fibrosis frequently remain clinically silent but portend adverse outcomes and reduced quality of life.

The rise of advanced noninvasive imaging modalities, including echocardiography, CMR, CCTA, and CAC scoring, has transformed the landscape of cardiovascular care in CKD. These techniques facilitate early detection and characterization of both cardiac and vascular disease, enabling clinicians to intervene before irreversible tissue injury or clinical decompensation occurs. When coupled with the expanding arsenal of injury biomarkers, clinicians are now equipped to risk-stratify, prognosticate, and personalize management for CKD patients long before conventional clinical endpoints are reached. Increasingly, serial and trend-based assessments are recognized for their superior predictive value in monitoring disease progression and guiding therapy. Closing the mortality gap will require a shift toward proactive care – prioritizing early recognition, individualized risk stratification, and multidisciplinary management – ultimately improving outcomes and quality of life for this vulnerable population.

**Table 1.** Advanced imaging modalities for detection of subclinical cardiovascular disease in chronic kidney disease

Modality	What it detects	CKD-specific considerations	Strengths	Limitations
Transthoracic Echocardiography (TTE)	LV mass/geometry, systolic, diastolic function, valvular disease, LA size, pulmonary pressures, global longitudinal strain (GLS)	Widely available; results influenced by volume status, anemia, and blood pressure changes common in CKD	Real-time functional assessment, serial monitoring, no contrast or radiation, 3D improves LV mass accuracy	Operator-dependent; acoustic window limitations
Stress Echocardiography	Inducible ischemia, contractile reserve	Useful for pre-transplant and symptomatic evaluation; exercise tolerance often limited in CKD	Good negative predictive value for obstructive CAD, no ionizing radiation	Reduced sensitivity with baseline LV dysfunction; limited by patient ability to exercise, image quality
Cardiac Magnetic Resonance (CMR)	Accurate volumes/mass, focal and diffuse fibrosis (late gadolieum enhancement, native T1, extracellular volume fraction, edema, tissue characterization)	Gadolinium Group II agents now considered low risk when clinically indicated – weigh diagnostic benefit vs. renal status; non-contrast mapping possible	Highest spatial resolution; reproducible LV mass and fibrosis quantification; non-contrast mapping detects diffuse fibrosis	Less available and more costly; longer exam time; gadolinium caution in advanced CKD
Coronary CT Angiography (CCTA)	Coronary anatomy, luminal stenosis, plaque burden and composition (calcified, noncalcified, low-attenuation plaque)	Iodinated contrast required – modern low-osmolar agents and hydration strategies reduce AKI risk; photon-counting CT can lower iodine dose	High negative predictive value for CAD; plaque characterization and high-risk plaque features; rapid acquisition	Requires contrast; susceptible to motion, arrhythmia artifacts; artifact in heavy calcification (common in CKD)
Coronary Artery Calcium (CAC) Scoring	Quantitative calcified plaque burden (Agatston score)	Very commonly elevated in CKD; high CAC predicts events but may reflect medial calcification as well as intimal atherosclerosis	Simple, no contrast, prognostic for CV events; useful risk stratification tool	Does not detect noncalcified plaque; interpretation influenced by age, race, and CKD-specific medial calcification

**Table 2.** Key biomarkers for identification of subclinical CVD in CKD

Biomarker category	Key biomarkers	Physiologic role	CKD-specific considerations	Relevance to subclinical CVD in CKD
Cardiac Injury Markers	High-sensitivity troponin (hsT); B-type natriuretic peptide (pBNP)	hsT: Detects myocardial injury. proBNP: Released in response to ventricular volume/pressure load	hsT chronically elevated due to reduced renal clearance; dynamic changes remain predictive. proBNP elevated with impaired excretion even without HF. Requires serial trend interpretation	Identify subclinical myocardial injury, remodeling, and volume overload; predict CV events, HF hospitalization, and mortality
Renal/Vascular Injury Markers	Urine albumin-to-creatinine ratio (UACR)	Reflects glomerular permeability, microvascular injury, and systemic endothelial dysfunction	Not significantly affected by reduced clearance; interpretable across CKD spectrum	Independent predictor of all-cause and CV mortality. Risk increases even within the “normal” UACR range. Integrates vascular and renal injury; refines CKD staging and prompts intensified prevention strategies
Inflammatory, Oxidative Stress Markers	CRP; IL-6; TNF-α	Reflect systemic inflammation and endothelial dysfunction	IL-6 and TNF-α often chronically elevated in dialysis due to impaired clearance and inflammatory milieu. Persistent elevations predict worse outcomes	Predict atherosclerotic progression, MI, vascular dysfunction, HF events, and mortality; reflect chronic inflammatory burden driving CVD in CKD
Novel Biomarkers	FGF-23; Soluble ST2 (sST2); GDF-15; Galectin-3; NGAL; KIM-1	FGF-23: mineral metabolism and LVH signaling. sST2, GDF-15, Galectin-3: myocardial fibrosis, remodeling, tissue stress. NGAL and KIM-1: tubular injury and AKI/CKD progression	Levels influenced by altered renal clearance and chronic inflammation; require stage-specific interpretation	Predict LVH, HF progression, all-cause mortality, and heightened atherosclerotic risk. Offer potential for early detection and personalized CVD risk assessment in CKD

NGAL, neutrophil gelatinase-associated lipocalin.

## Conflict of Interest Statement

Dmitry Abramov was a member of the journal’s Editorial Board at the time of submission and has received speaker fees from AstraZeneca and Bayer. The other authors have no conflicts of interest to declare.

## Funding Sources

The study was not supported by any sponsor or funder.

## Author Contributions

Inhae Baek, MD and Anas Alani, MD: writing–original draft and visualization. Inhae Baek, MD, Purvi Parwani, MD, Safwan Gaznabi, MD, Dmitry Abramov, MD, and Anas Alani, MD: review and editing.
